# Artificial intelligence in endocrinology: a comprehensive review

**DOI:** 10.1007/s40618-023-02235-9

**Published:** 2023-11-16

**Authors:** F. Giorgini, G. Di Dalmazi, S. Diciotti

**Affiliations:** 1 Department of Electrical, Electronic, and Information Engineering “Guglielmo Marconi”, University of Bologna, Cesena, Italy; 2 Division of Endocrinology and Diabetes Prevention and Care, IRCCS Azienda Ospedaliero-Universitaria di Bologna, Bologna, Italy; 3 Department of Medical and Surgical Sciences (DIMEC), Alma Mater Studiorum University of Bologna, Bologna, Italy; 4 Alma Mater Research Institute for Human-Centered Artificial Intelligence, University of Bologna, Bologna, Italy

**Keywords:** Adrenal tumors, Bone and mineral disorders, Diabetes disorders, Machine learning, Pre-emptive medicine, Risk prediction

## Abstract

**Background and aim:**

Artificial intelligence (AI) has emerged as a promising technology in the field of endocrinology, offering significant potential to revolutionize the diagnosis, treatment, and management of endocrine disorders. This comprehensive review aims to provide a concise overview of the current landscape of AI applications in endocrinology and metabolism, focusing on the fundamental concepts of AI, including machine learning algorithms and deep learning models.

**Methods:**

The review explores various areas of endocrinology where AI has demonstrated its value, encompassing screening and diagnosis, risk prediction, translational research, and “pre-emptive medicine”. Within each domain, relevant studies are discussed, offering insights into the methodology and main findings of AI in the treatment of different pathologies, such as diabetes mellitus and related disorders, thyroid disorders, adrenal tumors, and bone and mineral disorders.

**Results:**

Collectively, these studies show the valuable contributions of AI in optimizing healthcare outcomes and unveiling new understandings of the intricate mechanisms underlying endocrine disorders. Furthermore, AI-driven approaches facilitate the development of precision medicine strategies, enabling tailored interventions for patients based on their individual characteristics and needs.

**Conclusions:**

By embracing AI in endocrinology, a future can be envisioned where medical professionals and AI systems synergistically collaborate, ultimately enhancing the lives of individuals affected by endocrine disorders.

## Introduction

The exponential growth of technology in the past two decades has paved for the development of advanced techniques capable of addressing scientific inquiries at a magnitude far surpassing human capabilities. One notable example is the field of artificial intelligence (AI). AI is a branch of computer science that focuses on the theory and development of computer systems and algorithms capable of performing tasks that typically require human intelligence [1]. The healthcare sector is currently undergoing an unprecedented transformation due to AI, as it possesses the potential to enhance existing clinical practices. The innovative aspects introduced by AI find ideal applications within the field of endocrinology, given its complex and interconnected nature. Indeed, unlike other medical domains, endocrinology is not related to a single organ structure but is a complicated biological system of hormones and metabolites, where hormones function within an elaborate network of local and remote actions involving receptors, signaling pathways, and intricate feedback mechanisms [2, 3]. These complex and interconnected systems are often beyond the comprehension and reasoning abilities of the human brain. The purpose of this study is to explore the diverse applications of AI in the field of endocrinology and metabolism, with a focus on its potential to enhance screening, disease diagnosis, risk prediction, prognosis, and medical research. Consequently, it offers an overview of AI's capacity to gather valuable information, deliver personalized care, and enhance patient outcomes within the dynamic field of endocrine and metabolic diseases.

## Artificial intelligence: a brief introduction

The terms artificial intelligence, machine learning (ML), and deep learning (DL) are often used concurrently and sometimes interchangeably in medical literature. However, they are not synonyms. AI is the field that encompasses the theory and advancement of computer systems and algorithms designed to execute tasks that commonly necessitate human intelligence; ML is a subset of AI techniques, including those methods that use statistical learning to allow machines to improve with experience. DL, in turn, is a subset of machine learning techniques that use complex algorithms inspired by the human brain and how it works [4]. In the medical field, the class of algorithms that is being developed and used the most is ML. ML can be classified into four main categories: supervised, unsupervised, semi-supervised, and reinforcement learning as presented in Table 1 [5].

**Table 1:** Machine learning algorithms

Types of learning	Supervised learning	Unsupervised learning	Semi-supervised learning	Reinforcement learning
Concept	Learning a function that best approximates new input to the desired output based on a given relationship between the input and labeled output from the labeled dataset	Finding structures or patterns in an unlabeled dataset	A mixed approach of supervised and unsupervised learning applicable to a small amount of labeled data and a large amount of unlabeled data	Learning by maximizing the reward function based on the responses yielded by various actions to achieve arbitrary goals in a given unstructured or unknown environment
Common tasks	Regression, classification	Clustering, dimensionality reduction	Regression, classification	Taking actions to maximize the reward
Algorithms examples	k-nearest neighbors, linear/logistic regression, decision tree and random forest, support vector machines, neural network, etc.	K-means, expectation maximization, auto-encoders, principal component analysis (PCA), kernel PCA, etc.	Generative model, semi-supervised support vector machine, etc.	Q-learning, policy gradient, actor-critic, etc.

**Fig. 1:**
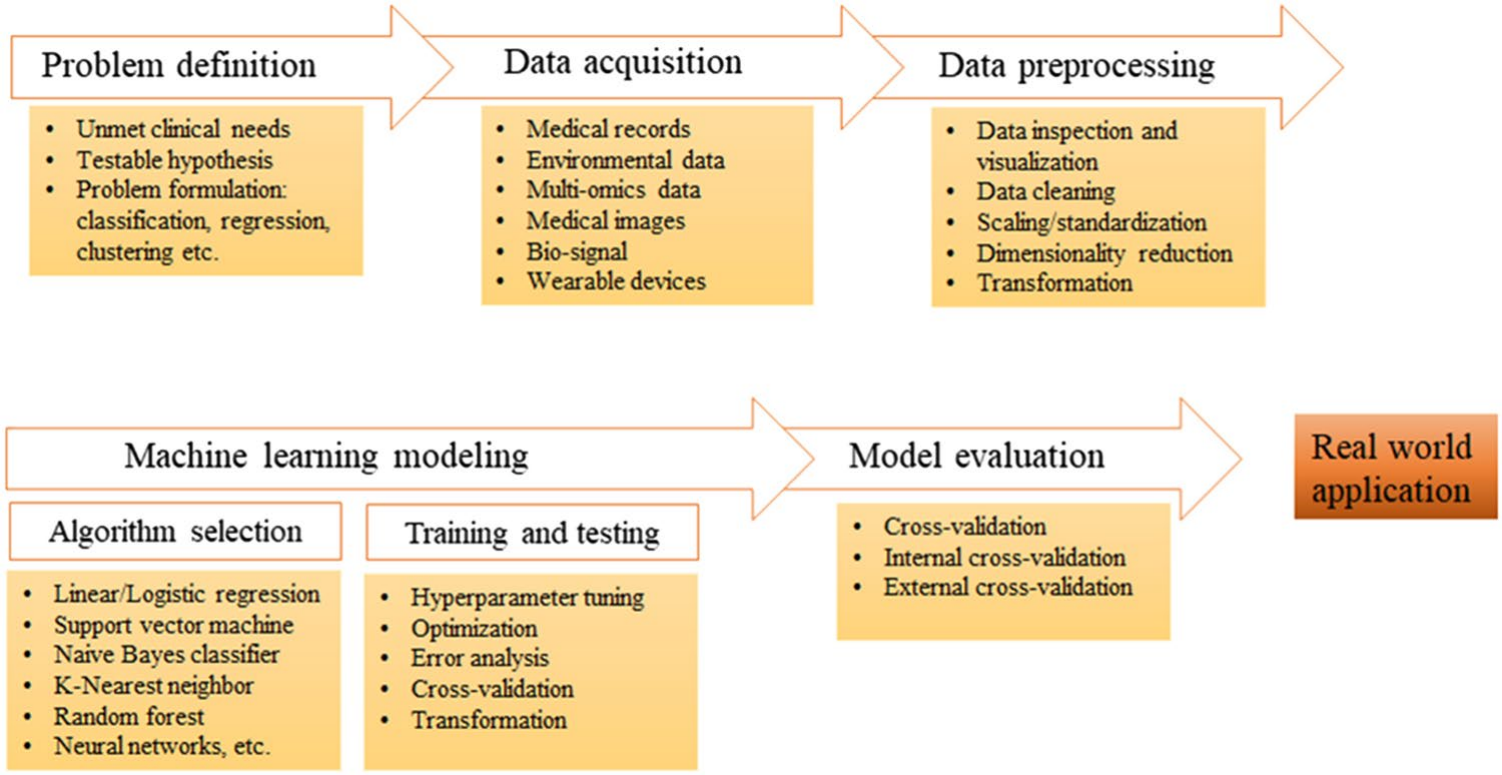
A brief workflow of machine learning-based medical research

Research investigations in the medical field related to AI, particularly based on ML, encompass a complex workflow. This workflow comprises problem formulation, data acquisition and preprocessing, the selection of a suitable ML model, and culminates in the comprehensive evaluation of the model (Fig. 1). It is crucial to emphasize that this workflow serves as a general outline and acknowledges that specific steps and details may vary depending on the research problem, available resources, and the requirements of the medical domain under study. For more comprehensive insights, refer to [5, 6].

## Methods

The comprehensive review we propose aims to achieve a concise overview of the landscape of AI applications in endocrinology and metabolism, with the goal of demonstrating its capability to collect valuable information, offer personalized care, and enhance patient outcomes. To achieve this objective, we employed a targeted search and selection strategy, which will be outlined in the following sections.

### Search strategy

F.G. conducted a comprehensive literature review by querying the PubMed database. Any disagreements were resolved by the senior reviewers S.D. and G.D.D. until an agreement was reached on all issues. Our search strategy involved a combination of specific keywords, including “machine learning”, “deep learning”, “pre-emptive medicine”, “diagnosis”, “prediction”, “risk prediction”, “detection” and “treatment” as well as Medical Subject Headings (MeSH) terms such as “diabetes”, “diabetes disorders”, “adrenal tumors”, “thyroid nodules”, and “bone and mineral disorders”. We connected these keywords and MeSH terms using “AND”, such as “machine learning” AND “diagnosis” AND “diabetes”, “machine learning” AND “prediction” AND “diabetes”, “machine learning” AND “detection” AND “diabetes disorders”, “pre-emptive medicine” AND “diabetes disorders” and so on. All conducted research was restricted to studies published from 2017 to the present, with a focus on articles available in full-text English. In this procedure, we also considered grey-literature sources, notably integrating selected proceedings [7, 8] and a report from a research institute [9] with the aim of elucidating the characteristics of specific diseases and highlighting the innovative aspects of “pre-emptive medicine”.

### Study selection

The study selection process was carried out in two stages: an initial screening of titles and abstracts, followed by a more detailed assessment of full-text articles. Eligible studies were included if they met the following three criteria: (1) ensuring an adequate number of subjects (n > 10) representing the variety of disease categories, including diabetes and related disorders, adrenal tumors, thyroid disorders, and bone and mineral disorders; (2) encompassing studies on ML and DL, with at least one study illustrating different ML applications such as supervised, unsupervised, and reinforcement learning; and (3) prioritizing studies with recent publication dates whenever possible. Subsequently, we classified these studies according to the domains in which AI plays a significant role in the field of endocrinology and metabolism. These domains include screening and diagnosis, risk prediction, translational research, and the emerging field of “pre-emptive medicine”, which may exhibit overlapping elements in certain cases. Furthermore, it is important to acknowledge that while these selected studies may not fully capture the entire spectrum of AI applications in the field of endocrinology, they provide practical examples that help illustrate the utility of ML and DL algorithms across various areas of endocrine research.

## Results

**Fig. 2:**
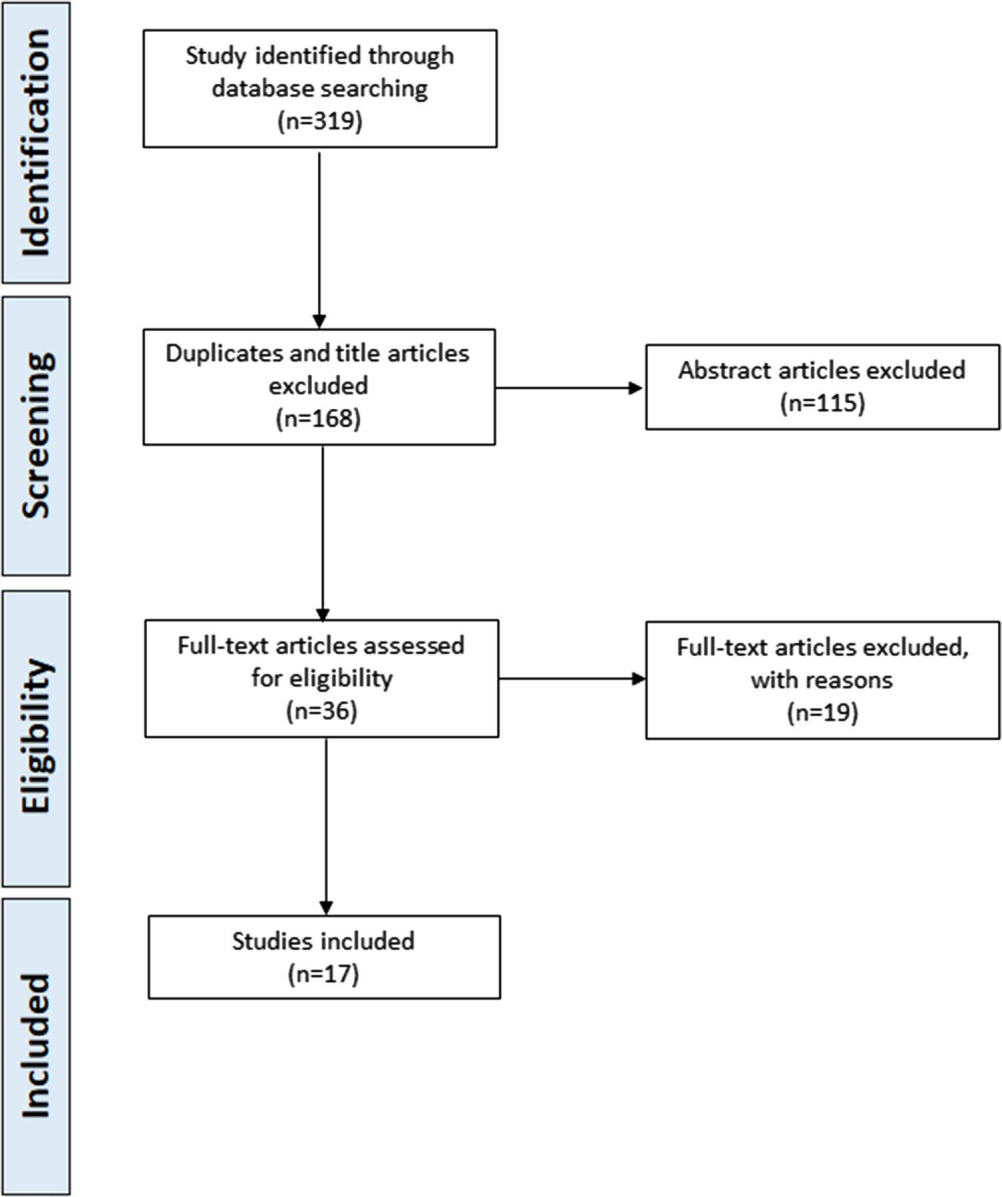
Flowchart of the included studies

Among the 319 articles that we initially identified, 168 were removed after evaluating the titles and removing duplicates. Among the remaining 151 studies, 115 were excluded after reviewing their abstracts, since they did not satisfy the inclusion criteria. We conducted a meticulous examination of the remaining 36 full-text articles. Ultimately, 17 articles [10–26] met the predefined inclusion criteria. The flowchart of the included studies is presented in Fig. 2 while the main features of the included studies are summarized in Table 2.

**Table 2:** Summary of studies related to AI applications in Endocrinology field

Task	Study (disease field)	Study subjects	Design and method	Principal results
Screening and diagnosis	Agliata et al. [10] (Diabetes and related disorders)	Three datasets	Aim: to investigate the correlations between an individual's health status and the development of type 2 diabetes in order to accurately predict its onset or assess the individual's risk level	The ablation study revealed that an ensemble of binary classifiers with a shallow architecture optimized using the Adam algorithm attained a satisfactory level of accuracy (approximately 86% on the test set) and an AUROC value of 0.934
		The NHANES including a sample of adult US citizens, aged 18 years or older, in the range of seven thousand individuals for each year		
		MIMIC-III containing anonymized health-related information on more than 40,000 patients who received ICU care at Beth Israel Deaconess Medical Center between 2001 and 2012	Methods: supervised ML: neural network	This neural network-based approach may provide accurate information for personalized medicine, making it a valuable resource for decision making
		MIMIC-IV is an upgrade to MIMIC-III that adds modern data and enhances many elements of the previous version		
		These datasets were combined to create a single dataset of 13,687 individuals with a similar number of individuals with and without type 2 diabetes		
	Raju et al. [11] (Diabetes and related disorders)	The Kaggle DR dataset composed of 88,702 colour fundus images, including 35,126 samples for training and 53,576 samples for testing	Aim: to classify the stage of DR and detecting the laterality of the eye using funduscopic images	High sensitivity (80.28%) and specificity (92.29%) in the detection of DR staging, as well as laterality of the eye from funduscopic retinal images using a CNN
			Methods: DL: CNN	CNNs have the potential to automatically classify fundus images based on laterality and severity in real time
	Liu et al. [12] (Adrenal tumors)	A total of 188 tumors were observed in the 183 patients with LPA, while 92 tumors were identified in 86 patients with sPHEO. The pre-enhanced CT imaging characteristics of these tumors were assessed	Aim: to assess the accuracy of CT-based machine learning models for differentiating sPHEO from LPA in patients with adrenal incidentalomas	The LR model performed better than other models
			Methods: supervised learning: LR, SVM and RF	The LR model (M1) including three CT features: CTpre value, shape, and necrosis/cystic changes had an AUROC of 0.917 and an accuracy of 0.864
				The LR model (M2) including three CT features: CTpre value, shape and homogeneity had an AUROC of 0.888 and an accuracy of 0.832
				The S2 scoring system (sensitivity: 0.859, specificity: 0.824) had comparable diagnostic value to S1 (sensitivity: 0.815; specificity: 0.910)
				Results indicated the potential of using a non-invasive imaging method such as CT-based machine learning models and scoring systems for predicting histology of adrenal incidentalomas
	Valentinitsch et al. [13] (Bone and mineral disorders)	CT data from 154 consecutive patients between February 2007 and February 2008	Aim: to identify individuals with vertebral fractures using opportunistic CT screening	The ML model, incorporating global and local density as well as texture parameters, demonstrated superior performance in identifying individuals with vertebral fractures when compared to relying solely on volumetric BMD (AUROC: 0.88 vs. 0.64)
			Methods: supervised ML: RF	Developed a quantitative and automated pipeline for opportunistic CT screening aimed at identifying individuals with vertebral fractures
	Somnay et al. [14] (Bone and mineral disorders)	Retrospective cohort of 6777 patients with confirmed primary hyperparathyroidism who underwent parathyroidectomy vs. 5033 controls who underwent thyroidectomy from March 2001 to August 2013	Aim: to establish an ML model discriminating patients with primary hyperparathyroidism among patients who underwent neck surgery	ML model helped identifying individuals with primary hyperparathyroidism among subjects who underwent neck surgery (accuracy 95.2%; 71.1% in mild case)
			Methods: supervised ML: naive Bayesian network with adaptive boosting	Tested algorithm performance in the context of various relevant clinical situations
	Peng et al. [15] (Thyroid disease)	Multicentre study that used ultrasound image sets from seven hospitals in China divided in	Aim: to develop a DL AI-assisted strategy for clinical decision-making regarding thyroid nodules	The model showed advantages in improving the accuracy of diagnosis, especially for junior radiologists
		Training set: 18,049 images of 8339 patients	Methods: DL: ThyNet (combined architecture of three networks: ResNet, ResNeXt, and DenseNet)	In the clinical setting test, sonographic assessment of thyroid nodules included both real-time dynamic nodule visualisation and interpretation of static images. ThyNet also improved the performance of radiologists in this clinical setting
		Test set A: 2185 images from 1424 patients		
		Test set B: 1754 images from 1048 patients		
		Test set C: 366 images from 303 patients		
				The ThyNet-assisted fine needle aspiration strategy could be useful for the avoidance of unnecessary invasive biopsy
	Perakakis et al. [16] (Diabetes and related disorders)	Serum samples of 49 healthy subjects and 31 patients with biopsy-proven NAFLD	Aim: to train models for the non-invasive diagnosis of NASH and liver fibrosis based on circulating lipids, glycans, fatty acids identified by LC–MS/MS and biochemical parameters	The ML model including 20 features consisted of lipidomics, glycans, and adiponectin yielded high accuracy up to 90% in discriminating healthy individuals from patients with NAFLD and NASH
			Methods: supervised ML: one-versus-rest nonlinear support vector machine models with recursive feature elimination	May provide a low-risk cost-effective, non-invasive alternative method to liver biopsy
	Cho et al. [17] (Diabetes and related disorders)	Four cohorts including those of the AA, HEXA, and CAVAS cohorts, which are part of the Korean Genome and Epidemiology Study cohort, and the KNHANES cohort Age, sex, and BMI information was obtained from the participants’ records A family history of diabetes was identified from a questionnaire	Aim: to identify distinct population clusters that exhibit variations in the development of type 2 diabetes Methods: unsupervised ML: RFC in discovery data; classification model to identify the clusters in the validation data: SVM	The prevalence of type 2 diabetes in the clusters increased as the risk factors became more saturated in the clusters Not only the distributions of 5 risk factors and prevalence were different between clusters, but the clusters also showed the significant difference of biochemical profiles as well Results indicates that diabetes-related metabolism might be heterogenous between clusters Results might be applicable to the study of precision medicine that aims to classify subpopulations according to differences in their susceptibility to a particular disease and the biology of that disease Population clusters might be able to develop more cost-effective method in the prevention of type 2 diabetes
	Marquadatd et al. [18] (Adrenal tumors)	RNA-sequencing data provided by the TCGA-ACC consortium consisting of 79 ACC samples ENSAT dataset containing RNA-sequencing results, consists of 7 ACC samples, but mainly of non-malignant forms: 4 normal adrenal glands and 52 adrenocortical adenomas, differentiating between endocrine inactive adenomas (9), adenomas with mild autonomous cortisol secretion (17) and Cushing syndrome cortisol producing adenomas (26)	Aim: to cluster adrenocortical tumors solely based on mRNA expression Methods: unsupervised ML: UMAP clustering; supervised RF classifier to specify the transcriptional differences between the two identified clusters	No limiting input data Found two clusters that match to a large extent (> 80%) the already published and well-known ACC clusters (C1A/C1B) Survival analyses confirmed the clusters found by the approach and show a significant survival advantage for the C1A cluster Examination of known mutations distribution within the clusters showed a significant accumulation of mutations of the *CTNNB1* and *TP53* genes in the poorer survival cluster C1B The use of a RF learning revealed the 100 genes that have the greatest influence on the separation of the two clusters and could potentially serve as new biomarkers or novel targets for therapeutic approaches
Risk prediction	Nicolucci et al. [19] (Diabetes and related disorders)	The dataset consists of 147,664 patients seen during 15 years from 23 Italian diabetes centers	Aim: to construct predictive models of DCs by big data machine learning, based on electronic medical records Methods: supervised ML: XGBoost	ML approach offers the opportunity to identify patients at greater risk of complications For all DCs considered, the predictive models in task 1 showed an accuracy > 70%, and AUROC largely exceeded 0.80, reaching 0.97 for nephropathy For all DCs considered, all predictive models for task 2 showed an accuracy > 70% and an AUROC > 0.85 Sensitivity in predicting the early occurrence of the complication ranged between 83.2% (peripheral vascular disease) and 88.5% (nephropathy)
	Jiang et al. [20] (Diabetes and related disorders)	Retrospective cohort of 1157 patients with type 2 diabetes with coronary plaque detected on CCTA at the West China Hospital from January 2018 and November 2021	Aim: to clarify the heterogeneity of coronary artery disease Methods: unsupervised ML K-prototypes algorithm with the elbow method	The clustering method could not only distinguish type 2 diabetes patients with different clinical contexts, but also indirectly identify the group with different types of coronary plaque Cluster 3 had relatively more segments with mixed and noncalcified plaques, Cluster 1 had the least number of obstructive coronary stenosis cases and the lowest proportion of patients with obstructive coronary disease in this study Although there were some differences in coronary plaque types and the degree of luminal stenosis, there was no significant difference in the extent of coronary atherosclerosis among the three cluster groups
	Oroojeni et al. [21] (Diabetes and related disorders)	Medical records of 87 patients with type 1 diabetes from Mass General Hospital; data for each patient’s visits over a 10-year period (training set) between 2003 to 2013; HbA1c, body mass index, activity level, alcohol usage status, insulin (Lantus) dose	Aim: to explore an effective reinforcement learning framework for determining the optimal long-acting insulin dose for patients with type 1 diabetes Methods: reinforcement learning; Q-learning with reward function set from HbA1c status at the visit and change of HbA1c from the past visit	The physician-prescribed insulin dose was within the dosing interval recommended by the Q-learning algorithm in 88% of test cases A proof-of-concept study to provide clinical decision support for determining insulin dose in patients with type 1 diabetes, by applying reinforcement learning algorithm
	Teh et al. [22] (Diabetes and related disorders)	43 consecutive patients with pDPN divided in: 29 responders defined as patients who report at least a 30% reduction in pain intensity score (0 to 10 numeric rating scale, where 0 = no pain and 10 = worse pain imaginable) post lidocaine treatment 14 non-responders	Aim: to classify treatment response in DPN using rs-fMRI and 3D-CNN deep learning architecture Methods: DL: 3D-CNN-Methods	Using ICA spatial component maps (ASC and SSC) performs better than only using RSP as the input to our CNN networks Using all the group ICA spatial components (ASC) information performs better compared to the semi-automatic selection of the highly relevant networks (SSC) A lightweight 3D-CNN deep learning architecture for classification uses imaging data more efficiently
	Zaborek et al. [23] (Thyroid disease)	Retrospective cohort of 598 patients who underwent total or completion thyroidectomy with pathology showing benign thyroid disease	Aim: to develop an ML-based levothyroxine dosing scheme after total thyroidectomy to achieve euthyroidism Methods: supervised ML: SVM, Bayesian recurrent neural network, decision trees, RF, ordinary least squares regression, Poisson regression, gamma regression, ridge regression, LASSO	The predictive accuracy of the dose-suggestion algorithm was modest (64.8%), which was better than standard weight-based dosing (51.3%) Provided an ML algorithm to suggest dosing scheme of levothyroxine after total thyroidectomy, with better accuracy across body mass index levels
Translational research	Liu et al. [12] (Diabetes and related disorders)	20 drug-naive individuals with prediabetes (discovery cohort) Determined exercise responders and non-responders after 12-week high-intensity exercise training Collected pre- and post-exercise period feces to analyze gut microbiota profile	Aim: to find an ML model for predicting exercise responsiveness determined from exercise-induced alterations in the gut microbiota Methods: supervised ML: RF	The ML model identified 14 microbiome species and 15 metabolites from human feces were able to predict exercise responsiveness (AUROC 0.75 in the validation set) Provide an example of applying ML principles to human-to-mice translational study based on microbiome dataset
	Williams et al. [25] (Miscellaneous)	Prospectively collected data from archived samples, clinical data, with approximately 85 million protein measurements in 16,894 participants from various cohorts including UK Whitehall II, Fenland, HUNT3, US Covance, HERITAGE Family studies	Aim: to develop plasma protein-phenotype models for 11 different health indicators (focusing on percentage body fat and incident cardiovascular events as outcomes) Methods: supervised ML: dimensionality reduction by false-recovery rate-corrected *P* values, proportional hazards elastic net models	The ML algorithm found proteins associated with body fat percentage (leptin, FABP, SFRP4) and CV events (gelsolin, antithrombin III, sTREM-1) Reveals the potential of ML algorithm application to find novel proteomics-based biomarkers in large-scale, well-established cohorts
“Pre-emptive medicine”	Itoh et al. [26] (Diabetes and related disorders)	No dataset subjects were used in this study as it was proposed from a theoretical point of view	Aim: to completely prevent the onset of hypertension by precisely predicting the elevation of blood pressure, even when an individual has normal blood pressure or is in the early stages of hypertension, and performing interventions to avoid the development of hypertension Methods theoretically proposed: DL	Theoretical results: By comprehensively evaluating alterations in the biological functions associated with hypertension over the life course, the current status of hypertension progression in each person can be precisely determined By examining the chronologically accumulated biological data, it can also predict the future course of hypertension in an individual

AA, ansan and ansung study; ACC, adrenocortical carcinoma; ASC, all spatial components; AUROC, area under the receiver operating characteristic curve; BMD, bone mineral density; BMI, body mass index; CAVAS, cardiovascular disease association study; CCTA, coronary computed tomography angiography; CNN, convolutional neural network; CT, computed tomography; CTNNB1, catenin beta-1; CV, cardiovascular; DC, diabetic complication; DL, deep learning; DPN, diabetic peripheral neuropathy; DR, diabetic retinopathy; ENSAT, european network for the study of adrenal tumors; FABP, fatty-acid-binding proteins; HbA1c, glycated haemoglobin; HERITAGE, health risk factors, exercise training and genetics; HEXA, health examinees study; HUNT3, the third Nord-Trodelag health study; ICA, indipendent component analysis; KNHANES, korean national health and nutrition examination survey; LASSO, least absolute shrinkage and selection operator; LC–MS/MS, liquid cromatography-mass spectrometry; LPA, lipid-poor adenoma; LR, logistic regression; MIMIC, medical information mart for intensive care; ML, machine learning; mRNA, messenger ribonucleic acid; NAFLD, nonalcoholic fatty liver disease; NASH, nonalcoholic steatohepatitis; NHANES, national health and nutrition examination survey; pDPN, painful diabetic peripheral neuropathy; RF, random forest; RFC, risk factor clustering; RNA, ribonucleic acid; rs-fMRI, resting state functional magentic resonance imaging; RSP, pre-processed resting state image data; sPHEO, subclinical pheochromocytoma; SFRP4, secreted frizzled-related protein, SSC, selected spatial components; sTREM-1, soluble triggering receptor expressed on myeloid cells-1; SVM, support vector machine; TGGA, the cancer genome atlas; TP53, tumor protein 53; UK, united kingdom; UMAP, uniform manifold approximation and projection; US, united states

## Screening and diagnosis

AI has revolutionized the field of screening and diagnosis, significantly improving the accuracy, efficiency, and effectiveness of medical assessments. In particular, AI aims to enhance screening strategies, given their significant clinical impact on endocrine disorders. Additionally, it aims to streamline the diagnostic workflow by analyzing extensive patient data, including medical records, imaging scans, and laboratory results, enabling faster, more precise, and efficient evaluations. Furthermore, AI is poised to discover novel disease clusters and associations by identifying previously unknown patterns and connections within complex medical data, thereby expanding our understanding of diseases themselves. To demonstrate the tangible impact brought about by AI in screening and diagnosis, we will present several applications and categorize them based on the specific objectives AI seeks to achieve.

### Improvement of screening strategies

Efficient screening tools for endocrine disorders have the potential to bring about significant clinical benefits. These benefits include improving the prognosis of individual patients by enabling earlier detection of diseases, as well as optimizing the allocation of public health resources through targeted focus on high-risk individuals and avoiding unnecessary testing in low-risk groups. In this context, researchers have been exploring the capabilities of ML and DL algorithms to determine whether they can offer a superior approach to screening for different endocrine diseases. For instance, multiple studies have demonstrated the application of AI in the field of diabetes and related disorders, illustrating its potential for early diagnosis and the development of therapeutic strategies aimed at preventing or postponing the onset of complications. Agliata et al. set out to use a supervised ML technique to investigate the correlations between an individual's health status and the development of type 2 diabetes, aiming to accurately predict its onset or assess the individual's risk level [10]. They proposed the implementation of a binary classifier with a shallow architecture, specifically a neural network, trained from scratch, to detect potential non-linear associations between the onset of type 2 diabetes and a collection of parameters derived from patient measurements. The conducted ablation study by the researchers revealed that the binary classifier, optimized using the Adam algorithm, achieved a satisfactory level of accuracy (approximately 86% on the test set) and a receiver operating characteristic (ROC) area under the curve (AUC) value of 0.934 in predicting the onset of diabetes in non-linear relationships with specific patient measurements. This neural network-based approach holds promise in delivering accurate information for personalized medicine, thereby serving as a valuable resource for decision-making. Furthermore, AI-based algorithms have been extensively utilized and validated for the diagnosis and classification of diabetic retinopathy (DR). Raju et al. proposed a DL model consisting of a convolutional neural network (CNN) based approach to classify the stage of diabetic retinopathy and detect the laterality of the eye using funduscopic images [11]. They utilized the publicly accessible Kaggle DR dataset composed of 88,702 color fundus images, including 35,126 samples for training and 53,576 samples for testing,  exhibiting a sensitivity of 80.28% and a specificity of 92.29% on the test set. Furthermore, the network was trained on 8810 images to detect the laterality of the eye, achieving an accuracy of 93.28% on the validation set consisting of 8816 images [11]. AI is also widely used in the field of distinguishing and differentiating adrenal tumors through the utilization of imaging techniques such as computed tomography (CT). Liu et al. proposed the application of supervised ML prediction models and scoring systems to differentiate between subclinical pheochromocytoma (sPHEO) and lipid-poor adenoma (LPA) [12]. Specifically, they employed logistic regression (LR), support vector machine (SVM), and random forest (RF) approaches to assess the accuracy of CT-based ML models in distinguishing sPHEO from LPA in patients with adrenal incidentalomas. The results demonstrated that the LR model outperformed the other models, achieving an AUC of 0.917 and an accuracy of 0.864. Medical image data holds the potential to offer relevant features that are well-suited for opportunistic screening of endocrine disorders. Valentinitsch et al. developed and trained a supervised ML model to detect prevalent vertebral fractures using non-fractured vertebral regions from CT scans performed for various reasons [13]. By incorporating global and local density and texture parameters, the ML model exhibited superior performance compared to relying solely on volumetric bone mineral density (BMD) in discerning the presence of vertebral fractures. These findings suggest the potential of a semi-automated pipeline for opportunistically screening individuals at high risk of fractures.

### Facilitation of the diagnostic workflow

The facilitation of the diagnostic workflow is a crucial element in modern healthcare, as timely and accurate diagnoses play a vital role in effective treatment and patient care. AI systems contribute to streamlining the diagnostic process by analyzing large volumes of patient data, thereby assisting the decision-making process and reducing diagnostic uncertainty. Peng et al. developed a DL model named ThyNet to aid in the diagnosis and management of thyroid nodules [15]. ThyNet utilized ultrasound image sets to differentiate between malignant and benign tumors, enabling a strategy for clinical decision-making. The results of this study showed advantages in improving the diagnostic accuracy of radiologists on thyroid nodule differentiation and could potentially decrease the number of unnecessary fine needle aspirations. In certain diseases, a well-validated and accurate non-invasive ML or DL model may have the potential to replace standard invasive diagnostic methods. For instance, the global prevalence of nonalcoholic fatty liver disease (NAFLD) is experiencing a rapid increase. However, invasive liver biopsy continues to be the gold-standard method for diagnosing both NAFLD and nonalcoholic steatohepatitis. In a study by Perakakis et al., a supervised method consisting of an SVM model was developed to classify NAFLD [16]. This model utilized features obtained from lipidomic, glycomic and liver fatty acid analysis of serum samples. To detect liver fibrosis, a concise exploratory model focused on ten lipid species achieved high accuracy (up to 98%). This suggests that a targeted lipidomic approach holds promise as a non-invasive alternative diagnostic tool. However, it is essential to validate the model further across diverse ethnicities and individuals with varying degrees of liver disease severity. AI holds significant promise in mitigating diagnostic uncertainty, particularly in challenging domains such as asymptomatic hyperparathyroidism. The identification of this condition proves challenging due to its subtle biochemical alterations and overlapping phenotype with primary osteoporosis and other rare mineral disorders [7]. In a study by Somnay et al., a supervised ML model, specifically a Bayesian network, was trained to recognize primary hyperparathyroidism among patients who underwent neck surgery, such as thyroidectomy or parathyroidectomy [14]. However, the model exhibited relatively low performance in detecting mild disease.

### Finding novel disease clusters and associations

Exploring novel disease clusters and associations offers valuable insights into the intricate network of biological pathways and interactions within the human body. In this regard, AI algorithms play a crucial role by analyzing extensive datasets from various sources, thereby revealing previously undiscovered connections between diseases that may have evaded traditional analytical approaches. These advanced algorithms have the ability to identify complex interactions, genetic variations, and environmental factors that contribute to the development and progression of diseases. Cho et al. proposed a method to identify distinct population clusters that exhibit variations in the development of type 2 diabetes [17]. At first, they employed a risk-factor-based clustering (RFC) approach, which involved hierarchically clustering the population using profiles of five established risk factors for type 2 diabetes: age, gender, body mass index, hypertension, and family history of diabetes. The RFC analysis successfully identified six population clusters in the discovery data, showing significantly different prevalence rates of type 2 diabetes within each cluster. After identifying the clusters in the discovery data, an SVM model was applied to validate the findings. The SVM model also identified six clusters in the validation data, further confirming the heterogeneity of type 2 diabetes prevalence across these clusters. Notably, beyond variations in diabetes prevalence, the identified clusters exhibited distinct clinical features, including variations in biochemical profiles, and demonstrated different prediction performances using the risk factors [17]. Furthermore, in this context, unsupervised learning can be a valuable tool for discovering novel clusters and associations within a given dataset. For instance, Marquardt et al. proposed the utilization of an unsupervised ML-based method to cluster adrenocortical tumors solely based on messenger ribonucleic acid (mRNA) expression [18]. Specifically, they employed a visual-based clustering method on the ribonucleic acid (RNA) sequencing data from a large cohort of adrenocortical carcinoma (ACC) patients obtained from The Cancer Genome Atlas (TCGA). This approach successfully classified the tumors into two distinct clusters, which were found to be correlated with patient survival outcomes. Applying the visual clustering method to a second dataset that included benign adrenocortical samples, the study further revealed that one of the ACC clusters exhibited closer proximity to the benign samples. This observation provided a potential explanation for the improved survival observed in this particular ACC cluster. Moreover, by employing ML techniques, the researchers identified novel potential biomarker genes with prognostic value for this rare disease. These genes exhibited significant differential expression across the distinct survival clusters and warrant further evaluation [18].

## Risk prediction

AI algorithms can analyze extensive datasets encompassing patient information, laboratory results, imaging data, and genetic profiles to generate accurate risk prediction models for various endocrine disorders. By considering many factors and their complex interactions, AI can identify individuals at higher risk of developing conditions such as diabetes, thyroid disorders, or adrenal diseases. Furthermore, AI-driven models can evaluate treatment responses and predict patient outcomes based on clinical data, lifestyle factors, and treatment protocols. These predictive models enable endocrinologists to tailor treatment plans, optimize medication dosages, and make informed decisions regarding therapeutic interventions. In order to show the concrete advancements facilitated by AI in risk prediction, the subsequent applications are presented and classified into two categories: assessment of clinical outcomes and assessment of treatment responses.

### Assessment of clinical outcomes

The ability to accurately predict clinical outcomes empowers healthcare professionals to adopt an individualized approach to treatment strategy and monitoring. Several investigations have been conducted, for example, to address this objective, focusing on diabetes complications (DCs) through the utilization of ML techniques. These techniques provide an opportunity to identify patients who are at a higher risk of experiencing complications. A study conducted by Nicolucci et al. [19] serves as a notable example in this field. This study has focused on six categories of DCs: eye complications, cardiovascular diseases, cerebrovascular diseases, peripheral vascular diseases, nephropathy, and diabetic neuropathy. They developed a supervised learning approach utilizing tree-based algorithms (XGBoost) to predict the occurrence of each complication within a span of 5 years (task 1), as well as separate predictions for early (within 2 years) and late (3–5 years) onset of complications (task 2). The results for all DCs demonstrated predictive models with an accuracy exceeding 70% and an AUC surpassing 0.80, reaching 0.97 for nephropathy in task 1. For task 2, all predictive models exhibited an accuracy above 70% and an AUC greater than 0.85. The sensitivity in predicting the early occurrence of complications ranged from 83.2% for peripheral vascular disease to 88.5% for nephropathy [19]. An additional example is illustrated in the detection of coronary artery atherosclerosis in individuals with type 2 diabetes mellitus [20]. This is achieved through the utilization of an unsupervised clustering analysis based on clinical factors, which aims to differentiate the population heterogeneity of type 2 diabetes and evaluate the differences in coronary atherosclerosis as evaluated through coronary computed tomography angiography (CCTA). This method exemplifies the capability to effectively address patients with heterogeneous clinical indicators and identify groups with different types of coronary plaque and degrees of coronary stenosis [20].

### Assessment of treatment responses

ML and DL principles can be applied to predict treatment responses among patients affected by the same pathology. Teh et al. introduced a novel approach employing DL to predict the treatment response in individuals suffering from painful diabetic peripheral neuropathy (pDPN) [22]. They used resting-state functional magnetic resonance imaging (rs-fMRI) to extract functional connectivity features by means of group independent component analysis (gICA). Subsequently, they developed an automated treatment response classification model using three-dimensional convolutional neural networks (3D-CNN) to effectively distinguish between responders and non-responders to lidocaine treatment, showing the potential of deep learning in accurately predicting treatment outcomes for pDPN patients. Moreover, efficient ML and DL models hold promising potential in offering guidance for dose adjustment, particularly among patients with chronic conditions. For example, a reinforcement learning (RL) algorithm was developed to aid in determining the optimal dosage of long-acting insulin for individuals diagnosed with type 1 diabetes, utilizing clinical data [21]. This study demonstrates that an RL algorithm can be employed to provide personalized insulin doses, ensuring sufficient glycemic control in patients with type 1 diabetes. However, further investigation involving a larger patient sample is required to validate these findings. Another compelling example is provided by Zaborek et al., who constructed a supervised ML model to facilitate levothyroxine dose adjustment following thyroidectomy [23]. Their findings revealed a notable enhancement in predictive accuracy compared to the prevailing weight-based dosing approach, thereby demonstrating a substantial improvement.

## Translational research

ML algorithms have become a crucial methodology in translational research with the rise of the multi-omics approach, which produces abundant datasets with numerous features to be accounted for. Liu et al. developed an ML algorithm that integrated baseline microbial signatures to identify crucial microbiota species and metabolites strongly associated with exercise responsiveness in humans [24]. They observed distinct patterns of exercise-induced alterations in the gut microbiota between human exercise responders and non-responders. Moreover, through fecal microbial transplantation from responders to mice, they demonstrated that the benefits of exercise on insulin sensitivity could be conferred. By employing a random forest algorithm, they selected 19 features, among species and metabolites, which exhibited significant differences between the exercise-responsive and non-responsive groups. These selected features, among the numerous microbiota species and metabolites investigated, hold potential as biomarkers for personalized responses to exercise [24]. Another study aimed to identify proteomics-based biomarkers associated with various health outcomes, such as percentage body fat, lean mass, current smoking, and the risk of developing cardiovascular complications [25]. To accomplish this, the researchers adopted a comprehensive approach by leveraging extensive community-based cohort databases and samples. Employing ML techniques, they successfully discovered a set of highly predictive proteins and developed corresponding models. However, it is important to note that the practical application and generalizability of these findings must be confirmed through long-term studies conducted in diverse populations [25].

## Pre-emptive medicine

"Pre-emptive medicine" is an emerging field that leverages AI technology with the potential for extensive future applications. Originating in Japan, this concept aims to accurately anticipate the onset and progression of diseases by utilizing genomic information, biomarkers, bioimages, and other biological data. Its goal is to provide therapeutic interventions for diseases at their early stages, even before symptoms manifest in individuals. The concept of pre-emptive medicine takes into account the time-course of a disease in each individual and strives to employ medical interventions to prevent disease progression. Non-communicable diseases such as hypertension or diabetes [9] are particularly suitable and promising targets for pre-emptive medicine [8, 26]. For instance, in the context of pre-emptive medicine in hypertension, the ultimate goal is to completely prevent the onset of the pathology by precisely predicting the elevation of blood pressure, even in individuals with normal blood pressure or at early stages of hypertension [26]. To accomplish this, it is crucial to detect abnormal fluctuations in blood pressure as the earliest manifestation of the disease in an individual. Using the DL method, this analysis identifies changes in various biological data points that lead to increases or variations in blood pressure. By examining the chronologically accumulated biological data, it can also predict the future course of hypertension in an individual.

## AI limitations in medicine

AI algorithms heavily rely on the quality and quantity of data they are trained on [27]. Inaccurate or biased data can lead to flawed predictions and diagnoses, potentially compromising patient safety and outcomes [28]. Moreover, the issue of data privacy and patient confidentiality remains a significant concern, as the utilization of sensitive medical data for AI training purposes must adhere to stringent ethical and regulatory standards [29–31]. Another limitation arises from the "black-box" nature of some AI models, particularly in DL [27, 32]. Understanding how these models arrive at specific decisions can be challenging, hindering their acceptance among medical professionals who require transparency and interpretability in clinical decision-making [33]. Additionally, the integration of AI tools into existing healthcare systems and workflows poses practical challenges, including compatibility issues, staff training, and the need for substantial financial investments [33]. Furthermore, the regulatory landscape surrounding AI in medicine is continually evolving, and navigating these regulations while ensuring patient safety and efficacy can be a complex endeavor [34]. AI systems must meet stringent validation and verification criteria before widespread adoption can occur. Lastly, while AI algorithms can significantly enhance clinical decision-making, they should always complement rather than replace human expertise [35, 36]. Indeed, maintaining a human-in-the-loop approach cannot be overstated, as medical professionals remain essential for contextual understanding and ethical decision-making.

## Conclusions

In conclusion, this study has explored the remarkable potential of AI in the field of endocrinology by providing diverse examples of its applications. Through advancements in ML and DL, AI has demonstrated its ability to enhance various aspects of endocrine research and clinical practice. Improved screening, disease diagnosis, risk prediction, personalized treatment, and patient management are among the valuable contributions AI offers for optimizing healthcare outcomes in endocrinology. The application of AI algorithms in analyzing complex data sets has opened up new ways for understanding the intricate mechanisms underlying endocrine disorders. Moreover, AI-driven approaches enable the development of precision medicine strategies, offering tailored interventions for patients based on their individual characteristics and needs. As AI continues to evolve, it holds immense promise for transforming endocrinology by enabling more accurate diagnoses, potentially reducing unnecessary investigations, improving patient outcomes, reducing healthcare expenditures, facilitating efficient digital storage of vast patient data, and contributing to advancements in our understanding and management of endocrine-related diseases. Embracing AI in endocrinology can lead to a future where medical professionals and AI systems work synergistically, ultimately improving the lives of individuals affected by endocrine disorders.

## Data Availability

Not applicable.
